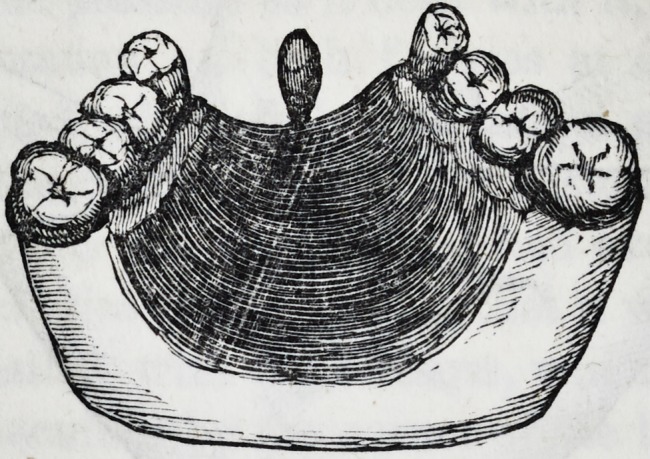# Double Congenital Hare-Lip—Absence of the Superior Incisors, and Their Portion of Alveolar Process

**Published:** 1844-09

**Authors:** J. Marion Sims

**Affiliations:** Montgomery, Alabama.


### ARTICLE V.

*Double Congenital Hare-lip—absence of the Superior Incisors, and their portion of Alveolar Process.* By J. MARION SIMS, M. D., Montgomery, Alabama.

Miss Margaret C., aged twenty-one, native of North Carolina, a resident of this state for the last two months, is the subject of this horrible deformity. The fissures extend entirely through the lip, into each nostril. That part of the lip, which, in its natural situation, would have formed the centre, is growing from the tip end of the nose, and is pushed forwards and upwards by a projecting bit of bone below it, which, on superficial examination, looks like a mere prolongation of the vomer, but it is, in reality, a misplaced portion of the alveolus. This bony *snout* is about three-fourths of an inch long, a little more than half an inch thick, rounded at its extremity; somewhat flattened laterally at its junction above with the septum nasi, and arched superiorly. In consequence of its exposure to the action of the atmosphere, its mucous covering is always dry. From its depending part, grew two teeth, which were extracted when she was about three years old, because they irritated the lower lip and kept it constantly sore. In the prolabrim of the lower lip are two depressions or *holes*, corresponding to the situation of the two teeth, which are perpetually secreting and pouring out a thin, glairy, viscid, translucent mucus.

The superior incisors and their portion of alveolus are wholly wanting. The palate is entire. Her voice is nasal and very unintelligible to one unaccustomed to it. She swallows fluids with great difficulty, as they regurgitate by the fissures in the lip and superior maxillare. This mortifying deformity has excluded her altogether from society. Modest, diffident and sensitive, she avoids the presence of every one, except her own brothers and sisters. Life has no charms for her; and her only solace here is her hope of a blessed immortality hereafter.

The accompanying cut is intended to represent, in some sort, the appearance of her deformity.

*a.* The end of the nose. *b.* The central piece of lip pushed forward by *c*, the bony process below. The lower lip with its two depressions, *ee.* The fissures extending up into each nostril.

In the performance of the operation it become a question whether the central piece of lip should be removed with the bony process or not. We, however, determined on attempting to force it down into its natural position to fill up the space between the lateral portions. Its distant removal from this place, seemed to forbid the effort, but then the scantiness of the side pieces, and the great extensibility of the part, appeared to justify it. On the ninth of June, 1842, in the presence of Drs. Colvin, Sloan and Bellangee, I dissected the central piece of lip from its bony companion for half an inch or more upwards and backwards, in the direction of the vomer, then passing the scalpel around the neck of the process, I divided its covering down to the bone and took it off with a pair of cutting pliers. After the cessation of the hæmorrhage, we forced the projecting tubercle of lip down, causing its raw surface to fit exactly over the cut end of the bone, and filling up perfectly the whole of the vacancy between the lateral portions. A strip of adhesive plaster, long enough to reach from one angle of the jaw to the other, two inches wide at each end, and but one-fourth of an inch wide in the middle, was applied, so that the middle narrow part pressed down the lip just at its junction with the septum, while the broad extremities were extended across the face in such a way as to support the cheeks and draw the lateral portions of lip towards the central.

The upper third of the central piece was pressed deeply and firmly immediately under the nose, the lower two-thirds hung loose, or with the mouth shut, was gently curled upwards by the protusion of the pouting lip below. This simple little strip of plaster was the only dressing used; and most elegantly did it answer the purpose. The fissures were so perfectly closed by it, that Miss C. was able for the first time in her life to enjoy the pleasure of a glass of water, without the usual unpleasant and mortifying attendance of regurgitation.

The dressing of adhesive plaster was removed as often as it seemed to slacken its hold. The wound between the lip and bone healed by the "first intention," and the operation might have been completed in five or six days, but it was deemed more prudent to keep up the constant pressure of the plaster, for a length of time, so as to mould the form of the part and accommodate it more accurately to its new and constrained position.

July 16th. As the central tubercle now occupies its place, just as well without the pressure as it does with it, we concluded to complete the operation on both fissures at once, in the usual way. The rounded edge of the left lateral portion of lip was cut according to the recommendation of Liston and others, with the knife. This was the first time I had ever used the knife, and I was so much dissatisfied with it that it was laid aside and the operation finished with the scissors, which I greatly prefer. By hooking a tenaculum by the corner of the lip, I could give it any degree of tension required, and with one stroke of the scissors divide the part quite into the nostril. Miss C., complained more of the pain inflicted by the knife than of that by the scissors. Perhaps the knife would have been less painful in the hands of a more dexterous operator. In spite of every entreaty she persisted in talking most vehemently, during the whole cutting process, which was, doubtless one, if not the principal cause, of the advantage that the scissors had over the knife. There was considerable hæmorrhage from the coronary arteries, which was very easily controlled by compressing the lip between the thumb and finger. After the bleeding ceased, a single needle was passed from side to side, transfixing the paterlous mouths of the coronarus, embracing both fissures with the central piece, and bringing the cut surfaces into exact opposition. At the upper end of each fissure and just within the nostril, the parts were brought together and secured by the "interrupted suture." The application of the suture so high within the nostril, was made easy by means of a very ingenious little needle, contrived by my excellent friend, Dr. William Bellangee, It has a straight shank, but is curved quite to a semi-circle at its point, much in the shape of a shepherd's crook, or the head of the fashionable walking canes of the day. An adhesive plaster, as before described, narrow in the middle and wide at each end, so as to support the cheek and prevent a stretching of the lip, completed the dressings.

Figure 2, is intended to show the appearance of the lip, with the dressing *in situ*, immediately after the operation.

July 23d. The dressings have not been disturbed till to-day, one week from the performance of the operation. The pin was blackened and rusty, it was a common sewing needle.

The sutures should have been removed sooner. The reason for allowing them to remain for such an unusual length of time was, that Miss C., got strangled in drinking some water on the third day after the operation, producing a very violent fit of coughing, which lasted, with scarcely an intermission, for nearly an hour. She was so exceedingly anxious about the consequences that she begged from day to day to put off the removal of the dressings, with the *hope* and belief that it would make the union more certain and secure. As no mischief was observed to result from their continuance, her earnest solicitude was indulged.

The cut surfaces adhered beautifully throughout their entire extent. She continued to wear a strip of adhesive plaster across the lip and cheeks for a month. The lip shows no notch or inductation in its border, but presents the appearance of a regular curved line. The point of the nose is slightly depressed, and the alae pushed a little outwards. I have wished very much to remedy this defect by a *third* operation, but she declares herself perfectly satisfied with her present condition, and altogether unwilling to undergo any more cutting.

However much Miss C's. *appearance* was improved by the operations on the lip, still they were unimportant in comparison with the *real* benefits derived from the operation of Dr. Bellangee, of this place, an ingenious, experienced and accomplished surgeon dentist.

Fig. 3. Is taken from a plaster cast in the doctor's possession, which exhibits the superior dental arch with the cuspid, two bicuspids and first molar on each side, the incisors and their alveolar ridge wanting. The projection in front is the vomer from which the piece of alveolus was cut in the first operation. This bit of bone projecting from the nostril and hanging over the vacant space in the superior maxillary, served an admirable purpose in giving support anteriorly to the artificial arch, which the doctor fashioned in his usually elegant and workman-like manner. The plate extended back as far as the space between the second bicuspid and first molar, fitting accurately the palatine arch and the necks of the intervening teeth, where it was substituted for the alveolar ridge, it not only supported four beautiful incisors, but it closed an opening which led from the mouth into the nostril, thereby preventing the secretions and fluids of one cavity from passing into the other.

## Figures and Tables

**Figure f1:**
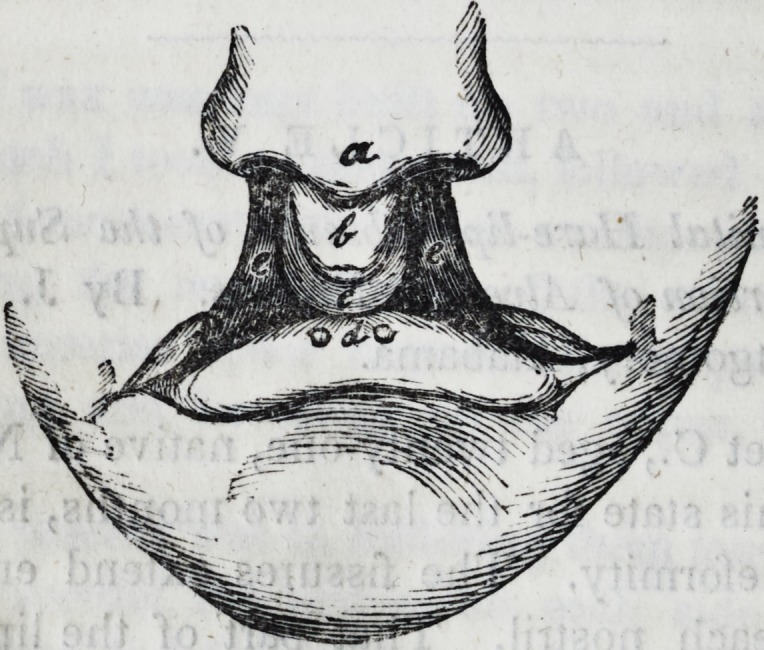


**Figure f2:**
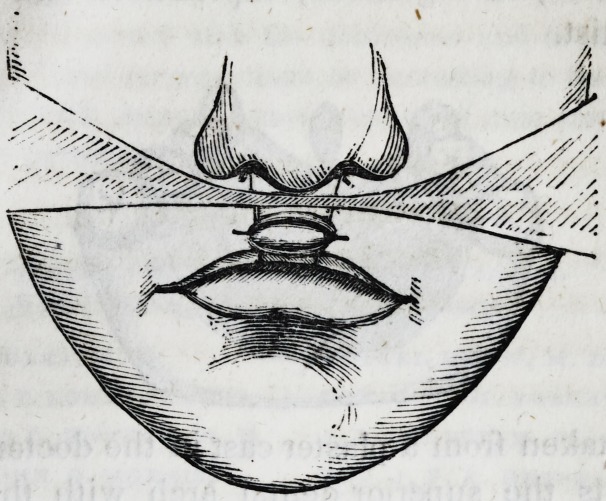


**Figure f3:**